# Lowering Caveolin-1 Expression in Human Vascular Endothelial Cells Inhibits Signal Transduction in Response to Shear Stress

**DOI:** 10.1155/2009/532432

**Published:** 2008-12-23

**Authors:** A. D. van der Meer, M. M. J. Kamphuis, A. A. Poot, J. Feijen, I. Vermes

**Affiliations:** ^1^Institute for Biomedical Technology and Department of Polymer Chemistry and Biomaterials, Faculty of Science and Technology, University of Twente, P.O. Box 217, 7500 AE Enschede, The Netherlands; ^2^Department of Clinical Chemistry, Medical Spectrum Twente, Hospital Group, P.O. Box 50000, 7500 KA Enschede, The Netherlands

## Abstract

Vascular endothelial cells have an extensive response to physiological levels of shear stress. There is evidence that the protein caveolin-1 is involved in the early phase of this response. In this study, caveolin-1 was downregulated in human endothelial cells by RNAi. When these cells were subjected to a shear stress of 15 dyn/cm^2^ for 10 minutes, activation of Akt and ERK1/2 was significantly lower than in control cells. Moreover, activation of Akt and ERK1/2 in response to vascular endothelial growth factor was significantly lower in cells with low levels of caveolin-1. However, activation of integrin-mediated signaling during cell adhesion onto fibronectin was not hampered by lowered caveolin-1 levels. In conclusion, caveolin-1 is an essential component in the response of endothelial cells to shear stress. Furthermore, the results suggest that the role of caveolin-1 in this process lies in facilitating efficient VEGFR2-mediated signaling.

## 1. Introduction

Vascular endothelial cells (ECs) are constantly subjected to shear stress caused by the flow of blood. ECs are highly responsive to changes in this shear stress. They are able to convert these mechanical stimuli into relevant biological signals by a process that is known as mechanotransduction [1]. The best-known elements in the early stages of this response are the cell-anchoring integrins [2] and certain membrane-associated receptors, such as the vascular endothelial growth factor receptor 2 (VEGFR2) [3, 4] and G-protein coupled receptors [5]. After the initial activation of these molecules, the biological signal is transmitted into the cell by activation of major signal transduction pathways, such as mitogen-activated protein kinase (MAPK) pathways, the protein kinase B (PKB/Akt) pathway, and the endothelial nitric oxide synthase (eNOS) signaling route. These events lead to a functional response of the cell, influencing rate of apoptosis and proliferation [1, 6], sensitivity to inflammation [7], and cytoskeletal remodeling [8].

Studies have shown that 50-nanometer, omega-shaped membrane invaginations, known as caveolae, are linked to mechanotransduction. The majority of membrane-associated proteins that are phosphorylated in response to shear stress localize to these domains [9, 10]. Also, as ECs are subjected to shear stress, the density of caveolae in the cell membrane increases, modulating the activation of signaling pathways [11, 12]. Moreover, mice that lack a structural protein of the caveolar domain, caveolin-1, have an abnormal vascular response when shear stress is altered [13].

In order to gain more mechanistic insight into the exact role of caveolae in the EC response to shear stress, in vitro studies were carried out to interfere directly with caveolar function. In some studies, caveolae were disrupted by cholesterol extraction [9, 14, 15], and in one study caveolar functioning was inhibited by introducing blocking antibodies to caveolin-1 [16]. These studies have shown that interfering with caveolar function causes an impaired response to shear stress, as characterized by a lowered activation of MAPKs [9, 14, 16], Akt [15], and eNOS [10].

An important molecular biological tool to study the role of proteins in cellular processes is RNA interference (RNAi). By transfecting cells with short interfering RNA (siRNA) molecules with a sequence that is complementary to the mRNA of the protein of interest, a dramatic lowering of the expression of this protein can be achieved. The tool is very specific and using it to lower expression levels of caveolin-1 could be useful in confirming the results obtained by the more crude method of cholesterol extraction.

In this study, we use RNAi in human umbilical vein endothelial cells (HUVECs) to confirm the essential role of caveolin-1 in EC mechanotransduction. We also show that lowering caveolin-1 levels leads to impaired VEGFR2 signaling, but not integrin signaling, in these cells. This suggests that the role of caveolin-1 in mechanotransduction lies in coupling VEGFR2 activation to downstream signaling.

## 2. Materials and Methods

### 2.1. Antibodies and Reagents

Caveolin-1 siRNA was bought from Qiagen (West Sussex, Ireland) (cat. no. SI00299635); negative control siRNA was obtained from Invitrogen (Calif, USA) (Stealth RNAi negative control with medium GC content). Antibodies for immunodetection were from the following companies: caveolin-1 (Sigma, Mo, USA; C4490), Akt (Abcam (Cambridge, UK); ab28422), phospho-Akt (Abcam, ab27773), ERK1 (Abcam, ab9363), phospho-ERK (Santa Cruz, Calif, USA; sc-7976), GAPDH (Abcam, ab9485), goat anti-rabbit IgG-Alexa 633 (Molecular Probes, Ore, USA; A21071), goat anti-rabbit IgG-HRP (Sigma, A0545), and mouse anti-goat IgG-HRP (Zymed, Calif, USA; 81-1620). All products for cell culturing were from Lonza (Breda, The Netherlands) except for the partially purified fibronectin, which was obtained as a coproduct during purification of human factor VIII at Sanquin, Amsterdam, The Netherlands, and which was used at 2 mg/mL in phosphate buffered saline (PBS) to coat surfaces for cell culture. All other reagents were from Sigma, except when specified differently.

### 2.2. HUVEC Isolation and Culturing

HUVECs were isolated from umbilical cords by the method of Jaffe et al. [17], using trypsin solution (0.05% (w/v) trypsin, 0.02% (w/v) EDTA in PBS). The obtained endothelial cells were cultured in fibronectin-coated culture flasks in endothelial growth medium-2 (EGM-2), containing 2% fetal bovine serum, until they reached confluency. When confluent, cells were detached from the surface using trypsin solution and diluted 1 : 3 in a fresh fibronectin-coated culture flask. Cells were kept in a humidified incubator (37°C, 5% CO_2_) up to passage 8, after which they were discarded.

### 2.3. siRNA Transfection

The day before transfection, HUVECs were seeded in a 6-well plate, 300⋅10^3^ cells per well in EGM-2. The following day, cells were washed once with OptiMEM medium (Gibco, Okla, USA) and then overlaid with 800 *μ*L OptiMEM. To transfect one well with siRNA, the following protocol was used. 15 *μ*L of 20 *μ*M siRNA solution were mixed with 145 *μ*L of 37°C OptiMEM medium and left at room temperature for 15 minutes. 8 *μ*L of Oligofectamine (Invitrogen) were mixed with 32 *μ*L OptiMEM, and after 5 minutes the Oligofectamine mixture was added to the tube with siRNA. Complexes were allowed to form for 15 minutes, and they were added to the cells. After 4 hours in the incubator, 500 *μ*L EGM-2 with three times the normal amount of bovine serum were added to the wells. After overnight incubation, the cells were washed with PBS and incubated in normal EGM-2 for 6 hours.

### 2.4. Cell Treatments

For shear stress experiments, four wells of siRNA-transfected cells were trypsinized and replated on a fibronectin-coated glass plate of 40 cm^2^. The cells were left to adhere for two hours in EGM-2, after which the medium was replaced by endothelial basal medium-2 (EBM-2) to starve the cells overnight. The next day, 48 hours after transfection, cells were subjected to shear stress in a custom-built parallel plate flow chamber. All parts of the setup were sterilized before use. The chamber consisted of two parallel glass plates, spaced 0.6 mm apart by glass spacers, held together by a stainless steel housing. The chamber was connected to a glass reservoir containing 200 mL shear medium (medium 199 with 100 units/mL penicillin and 100 *μ*g/mL streptomycin) and to a peristaltic pump (Watson-Marlow, Rotterdam, The Netherlands). The different parts of the setup were connected to each other in a closed circuit using silicone tubing (Versitec silicone, 5 mm inner diameter, Rubber BV, The Netherlands), and the entire setup was put in an incubator to maintain proper culturing conditions. Medium was then pumped through the flow chamber for 10 or 30 minutes at a rate of 200 mL per minute. This yields a theoretical estimate for the shear stress of approximately 15 dyne/cm^2^. After the treatment, the flow chamber was disassembled; the cells were washed once with PBS and scraped in 100 *μ*L ice-cold lysis buffer (1% (w/v) Triton X-100/PBS with added protease inhibitors (4-(2-aminoethyl)benzenesulfonyl fluoride, pepstatin A, E-64, bestatin, leupeptin, and aprotinin) and phosphatase inhibitors (microcystin LR, cantharidin, (−)-p-bromotetramisole, sodium vanadate, sodium molybdate, sodium tartrate, and imidazole)). Then, the lysate was incubated on ice for 15 minutes and centrifuged at 12000 × g for 10 minutes. The supernatant was transferred to a fresh tube, and the protein concentration was determined using a bicinchonic acid assay (Pierce, Etten-Leur, The Netherlands). An equivolume of 2× Laemmli sample buffer was added to the lysate. The resulting sample was boiled for 5 minutes and then stored at −20°C until use.

For VEGF stimulation experiments, siRNA transfected cells were incubated overnight in growth medium and then starved for 6 hours with EBM-2. Subsequently, 48 hours after transfection, the cells were washed once with PBS, followed by addition of EBM-2 with 200 ng/mL VEGF_165_. Cells were put in the incubator for 10 minutes, after which a lysate was prepared as described above.

The integrin activation experiments were performed as follows. After siRNA transfection, cells were starved overnight in EBM-2. The following day, 48 hours after transfection, cells were trypsinized, spun down, and resuspended in 2 mL EBM-2 with 2% (w/v) bovine serum albumin (BSA). The cells were kept in suspension in the incubator under constant, light agitation for 30 minutes. Then, 1 mL of the cell suspension was plated on a fibronectin-coated surface, while the remaining cells were spun down, washed once with PBS, and then resuspended in lysis buffer. The plated cells were left to adhere for 20 minutes, after which a lysate was prepared as described above.

### 2.5. Western Blot and Immunodetection

Samples were subjected to sodium dodecyl sulphate (SDS) poly(acrylamide) gel electrophoresis, Western blotting, and immunodetection according to common protocols. Shortly, samples were separated by size on a 10% (w/v) poly(acrylamide) gel, and the protein band pattern was transferred to a poly(vinylidene difluoride) membrane, using a Bio-Rad Mini-Protean 3 system. The membrane was blocked with 1% (w/v) nonfat dry milk in 25 mM Tris, 150 mM NaCl, 0.05% (v/v) Tween-20, pH 8.3 (TBS-T). The primary antibodies were applied to the membranes in blocking buffer, overnight at 4°C. After washing the membrane with TBS-T four times for 20 seconds and one time for 15 minutes, the secondary antibody was applied to the membrane in blocking buffer for one hour at room temperature. The same washing regime was repeated, and the membrane was overlayed with SuperSignal West Femto substrate (Pierce). After three minutes of incubation, the chemiluminescent signal was detected with a Kodak Image station with CCD camera.

### 2.6. Confocal Microscopy

Cells were seeded at a density of 150 · 10^3^ cells per well on fibronectin-coated glass coverslips in a 12-well plate. They were transfected with siRNA as described earlier, but with half the reagents per well. After transfection, the cells were incubated overnight in EGM-2, then washed with PBS, and fixed with 4% (w/v) formaldehyde/PBS for 15 minutes at room temperature. The coverslips were then covered with permeabilization buffer (PBS with 1 mg/mL BSA and 0,1% (w/v) Triton X-100) for 10 minutes. Primary antibodies were diluted in permeabilization buffer and then incubated on the coverslips for 1 hour at 37°C. The coverslips were washed three times for 5 minutes with PBS and were subsequently covered with the secondary antibody in a mild permeabilization buffer (PBS with 1 mg/mL BSA and 0.05% (w/v) Triton X-100). After incubating for 1 hour at 37°C, coverslips were washed and a 100 ng/mL solution of 4′,6-Diamidino-2-phenylindole (DAPI) was applied for 5 minutes to stain nuclei. After three more washes with PBS, coverslips were mounted on microscope slides using Mowiol (CalBiochem, Darmstadt, Germany) and stored at 4°C in the dark until they were imaged with a Zeiss LSM 510 confocal microscope. An estimate of the average staining intensity was performed by dividing the total signal in a field by the number of nuclei, as determined by using ImageJ image analysis software [18].

### 2.7. Statistical Analysis

Signal intensities of immunodetection on Western blots were quantified using ImageJ. The intensity of the specific signal was normalized to total protein content, as assessed by the loading control in the same lane. In order to compare these signal intensities between different experiments, the ratios between the intensity level of a sample and the total intensity of all samples in that experiment were determined. The averages of these normalized ratios were plotted, with the error bars representing standard deviation. Differences between these means were tested for statistical significance by performing an unpaired, two-tailed Student's *t*-test. Differences were considered to be statistically significant at *P*-values smaller than .05.

## 3. Results

### 3.1. Mechanotransduction in ECs

**Figure 1:**
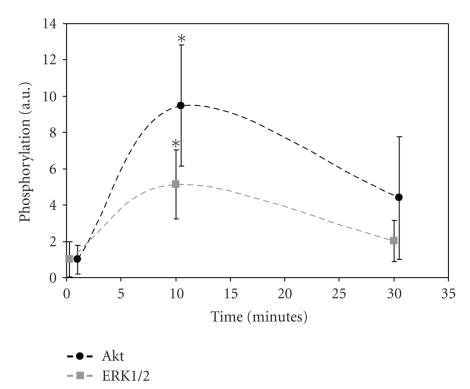
Activation of mechanotransduction pathways by shear. HUVECs were subjected to shear stress for different periods. Subsequently, the amount of phosphorylated Akt and ERK1/2 was determined by immunoblotting. After 10 minutes, phosphorylation levels were significantly higher (*P < .01, Student's *t*-test) compared to levels in statically cultured cells. After 30 minutes, this significant increase was no longer detected.

In order to investigate mechanotransduction in our flow chamber system, HUVECs were subjected to physiologically relevant shear stresses for 10 minutes and 30 minutes. After these treatments, cell lysates were tested for phosphorylated Akt and ERK1/2 by immunoblotting. As shown in Figure 1, 10 minutes of shear stress led to significantly enhanced phosphorylation of both Akt and ERK1/2. After 30 minutes of shear stress, phosphorylation levels had dropped to values comparable to the static situation. Therefore, we decided to focus on the early timepoint for our studies into the role of caveolin-1 in the EC mechanotransduction.

### 3.2. Caveolin-1 Downregulation

**Figure 2:**
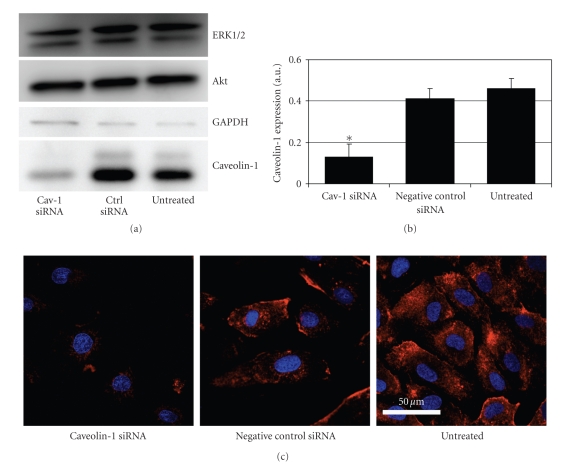
Downregulation of caveolin-1 expression by RNAi. HUVECs were treated with caveolin-1 siRNA, negative control siRNA, or left untreated. Caveolin-1 levels were determined 48 hours after transfection. (a) Immunoblot analysis of cell lysates. Equal loading of proteins of each transfection condition was ensured by detecting the unrelated metabolic enzyme GAPDH on the same blot. (b) Quantification of the signal intensities in the immunoblot assay. The bars represent average intensities of the caveolin-1 band in seven separate experiments. Error bars represent standard deviation. Caveolin-1 siRNA treated cells had significantly lower caveolin-1 expression than control siRNA-treated cells (*P < .0001, Student's *t*-test). No significant differences were found between untreated cells and negative control siRNA-treated cells. Bars represent averages of seven separate experiments. (c) Confocal microscopic imaging of caveolin-1. Treated cells were fixed and labeled with caveolin-1 antibodies that were detected by Alexa 633-coupled secondary antibodies. Nuclei were stained with DAPI. All images were taken with the same microscope settings. Scale bar is 50 *μ*m.

When HUVECs were treated with caveolin-1 siRNA, the expression of caveolin-1 decreased significantly to less than one-third of the caveolin-1 level in untreated cells, as shown by immunoblotting (see Figures 2(a) and 2(b)). When cells were transfected with negative control siRNA, no statistically significant decrease in caveolin-1 expression was detected, showing the specificity of the siRNA treatment. Moreover, expression of Akt and ERK1/2 was not affected by the transfection procedure or the lowering of caveolin-1 levels (see Figure 2(a)). The downregulation of caveolin-1 was confirmed by using immunocytofluorescence (see Figure 2(c)). Using the same microscope settings, staining intensity was approximately four times higher in untreated cells than in caveolin-1 siRNA-treated cells. The remaining caveolin-1 in the siRNA-treated cells was located mostly in the perinuclear area, with a lack of intense membrane staining like in the untreated and control RNA treated cells.

### 3.3. Mechanotransduction in ECs with Lowered Caveolin-1 Levels

**Figure 3:**
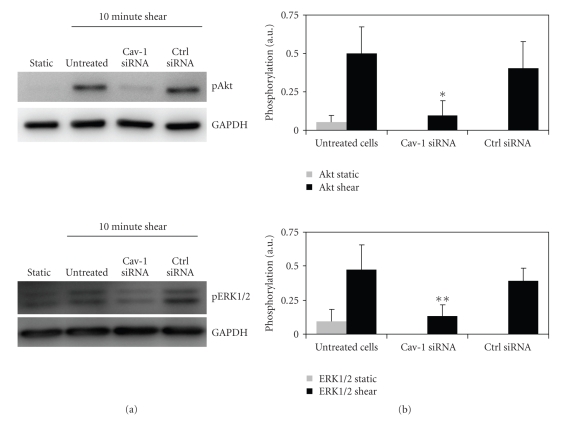
Activation of Akt and ERK1/2 in response to shear stress. HUVECs were transfected with either caveolin-1 siRNA, negative control siRNA, or left untreated. 48 hours after transfection, cells were subjected to a shear stress of approximately 15 dyn/cm^2^ for 10 minutes. (a) After treatment, cells were lysed, and the amount of phosphorylated Akt and ERK1/2 was determined by immunoblotting. Equal loading of proteins of each transfection condition was ensured by detecting the unrelated metabolic enzyme GAPDH on the same blot. The phosphorylation status of statically cultured cells is shown as a reference. (b) Quantification of the signal intensities in the immunoblot assay. Each bar represents the average intensity of four separate experiments. Error bars denote standard deviation. Akt and ERK1/2 phosphorylations increase five- to tenfold when untreated cells are subjected to shear stress. Both Akt and ERK1/2 phosphorylations are significantly lower (*P < .05, **P < .01, Student's *t*-test) when cells are treated with caveolin-1 siRNA instead of negative control siRNA prior to subjecting the cells to shear stress. No significant difference was detected between untreated cells and cells transfected with negative control siRNA, showing that the transfection procedure itself has no influence. Graphs represent the averages of four separate experiments.

HUVECs with normal and lowered levels of caveolin-1 were subjected to physiological levels of shear stress in a parallel plate flow chamber. After 10 minutes of shear stress, phosphorylation status of the important mechanotransducing molecules Akt and ERK1/2 was determined by phospho-specific immunoblotting. Total expression levels of these proteins were not affected by siRNA treatment, as determined by immunoblotting (see Figure 2(a)). After subjecting HUVECs to shear stress, phosphorylation status of Akt and ERK1/2 increased approximately five to ten times (see Figure 3). We found that the activation of both signal transduction pathways was significantly lower in caveolin-1 siRNA-treated cells than in cells treated with negative control siRNA. No significant differences were found among the activation levels of control siRNA-treated cells and untreated cells.

### 3.4. Signal Transduction in ECs with Lowered Caveolin-1 Levels

**Figure 4:**
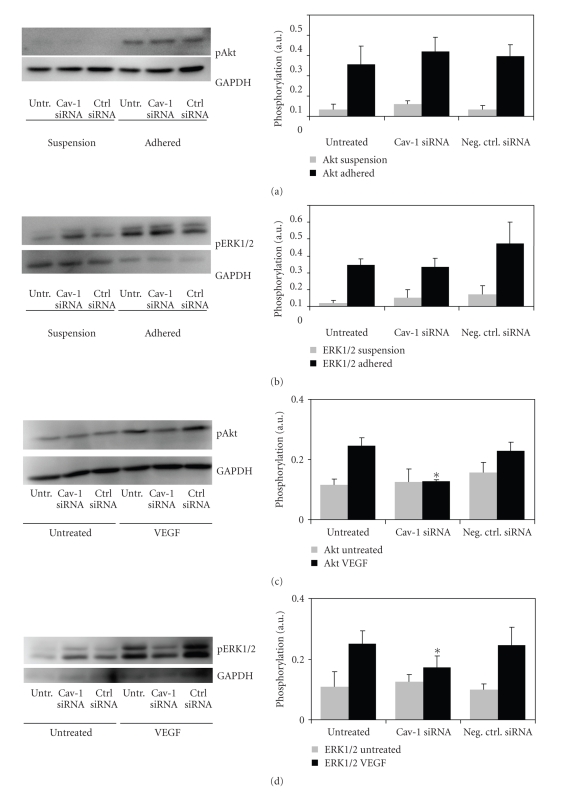
Activation of Akt and ERK1/2 in response to external stimuli. HUVECs were transfected with either caveolin-1 siRNA, negative control siRNA, or left untreated. 48 hours after transfection, cells were subjected to the following stimuli. (a), (b) Cells were kept in suspension for 30 minutes and then plated on fibronectin-coated culture plates to activate integrin-mediated signaling. Phosphorylation of both (a) Akt and (b) ERK1/2 increased significantly when compared to cells in suspension. No significant differences in activation levels were detected between HUVECs with high or low caveolin-1 levels. (c), (d) Cells were starved in basal medium and then treated with 200 ng/mL VEGF for 10 minutes. Phosphorylation of both (c) Akt and (d) ERK1/2 in response to this stimulus was significantly lower in cells with low levels of caveolin-1 than in cells that were treated with negative control siRNA (*P < .01). All bars are averages of three separate experiments.

In order to uncover the mechanism for the ineffective mechanotransduction in cells with low caveolin-1 levels, we induced signal transduction by activating cell surface receptors. We chose activation of integrins and VEGFR2, because these proteins are well known to be essential for mechanotransduction. In order to assess integrin-mediated signal transduction, cells were kept in suspension for 30 minutes and then plated for 20 minutes on a fibronectin-coated surface. When checking the phosphorylation status of Akt and ERK1/2 by phospho-specific immunoblotting, no significant differences could be detected between the activation of signal transduction pathways in negative control siRNA-treated cells and in cells with low caveolin-1 levels (see Figures 4(a) and 4(b)). Activation of VEGFR2 was accomplished by stimulating starved cells with 200 ng/mL VEGF_165_ for 10 minutes. In this case, signal transduction was found to be significantly lower in cells that had been treated with caveolin-1 siRNA than in cells with normal levels of caveolin-1 (see Figures 4(c) and 4(d)).

## 4. Discussion

Our study shows that lowering the expression level of caveolin-1 in human ECs by RNAi inhibits mechanotransduction in response to shear stress. Thus, we confirm the essential role of caveolae in the EC response to shear stress which has also been suggested by research groups that interfered with caveolar function using methods like cholesterol extraction [14] and antibody blocking [16].

We show that the activation of both Akt and ERK1/2 is affected by the lowering of caveolin-1. In both cases, this lowering will have functional repercussions for the cells. Akt is well known for its role in cell survival and resistance to apoptosis. Indeed, activation of Akt in response to shear stress has been shown to suppress apoptosis in ECs [15, 19]. Based on the findings in this study, one would expect that ECs with low caveolin-1 levels are more sensitive to apoptosis when subjected to shear stress than untreated cells. This would be an interesting and counterintuitive result, because lack of caveolin-1 generally renders cells less sensitive to apoptosis [20]. The exact mechanisms of this decreased apoptosis sensitivity have not yet been elucidated, although the Akt pathway is definitely involved [20]. A number of important membrane receptors in ECs, such as activin receptor-like kinase 1 (ALK-1) [21], VEGFR2 [22], and epithelial growth factor receptor (EGFR) [23], have been shown to associate with caveolin-1. Moreover, all mentioned receptors have been shown to induce Akt signaling [24–26]. Therefore, loss of the association between these receptors and caveolin-1 by downregulation of the latter may be a reason for changes in Akt activation and apoptosis sensitivity. Given the importance of EC apoptosis in the onset and development of atherosclerosis [27], future studies should be performed to provide more insight in this functional implication of the substantially lowered Akt activation in cells with less caveolin-1.

Another well-known downstream effect of Akt activation is increased eNOS activity [28]. Based on our finding that lowered levels of caveolin-1 inhibit activation of Akt, it can be expected that eNOS activation and the resulting effects on blood vessels, such as vasodilatation, are also impaired. Ex vivo studies have indeed shown that eNOS activation and vasodilatation in response to fluid flow are lower in arteries of caveolin-1 knockout mice than in blood vessels of wild-type mice [13].

The dramatically lowered activation of ERK1/2 in response to shear stress in caveolin-1 siRNA-treated ECs that we describe in this study will also have functional implications. Activation of ERK1/2 has an important role in modulating gene expression in response to shear stress. Two well-described examples show that ERK1/2 activation is essential for the shear stress-induced increase in the expression of matrix metalloprotease-9 [29] and eNOS [30]. The absence of ERK1/2 activation in our system will surely have an impact on these shear stress-induced changes in expression.

When attempting to identify the mechanism by which caveolin-1 contributes to mechanotransduction, we first decided to investigate the signal transduction in response to integrin binding in these cells. Integrin-mediated signaling is an essential process in mechanotransduction [2, 31]. Moreover, there have been multiple reports of the involvement of caveolin-1 in this signaling pathway [32–34]. Despite these reports, we were unable to detect any changes in activation of Akt or ERK1/2 between caveolin-1 siRNA-treated cells and untreated cells. It could be that in our system the remaining caveolin-1 is still sufficient to properly induce these signaling events. Our system differs from the ones used in the aforementioned reports by cell type, caveolin-1 levels, and the chosen output parameters to determine efficient signaling. Based on our experiments, we can conclude that it is unlikely that the inefficient mechanotransduction as seen in our system is due to impaired integrin signaling.

We decided also to investigate signal transduction in response to VEGF in caveolin-1 siRNA-treated ECs. Ligand-independent activation of VEGFR2 is another essential event in mechanotransduction [3], and stimulation with VEGF can serve as a way to specifically activate and monitor this part of the signaling network. The role of caveolin-1 in VEGF-induced signal transduction is still ambiguous and may depend on cell type and culture conditions. In some cell systems, downregulation of caveolin-1 leads to hyperactivation of signal transduction pathways [35]. In addition, other studies that focus on the role of caveolin-1 in VEGF-initiated signaling have shown that both an increase and a decrease of the levels of caveolin-1 in ECs have a negative impact on downstream signal transduction [22, 36]. The direct association between caveolin-1 and VEGFR2 inhibits activation of the latter, but on the other hand, the association also seems necessary for proper initiation of downstream signaling after growth factor receptor activation. Moreover, caveolin-1 knock-out mice have impaired vasculogenic potential, due to impaired VEGF signaling [36]. Also in our system, lowering the level of caveolin-1 has a negative impact on signal transduction when ECs are stimulated with VEGF. This suggests that the lack of mechanotransduction in ECs with low caveolin-1 levels is due to the important role that caveolin-1 plays in coupling VEGFR2 activation to downstream signaling events.

 Based on the results presented in this article, it can be concluded that caveolin-1 is needed for activation of Akt and ERK1/2 in ECs that are subjected to shear stress. Our results suggest that the impaired activation of these pathways in ECs with low levels of caveolin-1 is due to inefficient VEGFR2 signaling. This study highlights the importance of caveolin-1 in normal EC functioning. The role that caveolin-1 plays in the biology of ECs and the impact it has on the physiology of the vasculature in vivo are becoming more and more clear. Because of the importance of caveolin-1 in endothelial signaling, it is a potential target for clinical applications, for example, in tumor angiogenesis and atherosclerosis. Therefore, the role of caveolin-1 in the EC shear stress response is definitely worth studying in more detail.
